# Inhibition of *de novo* Methyltransferase 3B is a Potential Therapy for Hepatocellular Carcinoma

**DOI:** 10.4021/gr2008.10.1240

**Published:** 2008-11-20

**Authors:** Hong Fan, Jian Cheng, Zhu Jiang Zhao

**Affiliations:** a Key Laboratory of Developmental Genes and Human Disease, Ministry of Education, Southeast University; Department of Genetics and Development, Southeast University Medical School. 87 Dingjiaqiao, Nanjing 210009, Jiangsu Province, China

**Keywords:** Epigenetic, therapy, DNMTs, Hepatocellular Carcinoma, 5-Aza-CdR, siRNA, CpG islands, methylation

## Abstract

**Background:**

Aberrant epigenetic patterns, including inactivation of tumor suppressor genes due to DNA methylation, have been described in many human cancers. Epigenetic therapeutic is a new and rapidly developing area of tumor treatment because DNA methyltransferase (DNMT) inhibitors can reverse its changes. We attempted to identify potential approach for epigenetic therapy of hepatocellular carcinoma.

**Methods:**

We knocked down the expression of DNMT 1 or DNMT 3B by siRNA, and inhibited DNA methyltranferases by 5-Aza-2’-deoxycytidine. We used high-density oligonucleotide gene expression microarrays to examine the induced genes in human hepatocellular carcinoma cell line SMMC-7721 after suppressing DNA methyltranferases. The 5’ ends of up-regulated genes were analyzed by BLAST database to determine whether they have promoter CpG islands, and then the identical induced genes were compared among different inhibition of DNA methyltranferases.

**Results:**

Our results show that 9 genes were found to be over expressed by more than two-fold induced by DNMT1 siRNA and 5-Aza-CdR, and 30 genes were found to be over expressed by more than two-fold induced by DNMT3B siRNA and 5-Aza-CdR in SMMC-7721. Among them, 76.6% up-regulated genes conjectural contained 5’ CpG islands. The DNMT3B siRNA could induce more genes identical to demethylation agent in SMMC-7721.

**Conclusions:**

DNMT3B might be a new potential target for therapy of hepatocellular carcinoma.

## Introduction

Cancer is an epigenetic disease due to aberrant epigenetic patterns in the development of human cell, especially in abnormal DNA methylation. Transcriptional silencing of tumor-suppressor genes by CpG methylation may contribute to tumorigenesis. Moreover, the potential way is to drug-induced reactivation of methylation silenced tumor suppressor genes. 5-Aza-2’-deoxycytidine (5-Aza-CdR) inhibits DNA methylation and often is used *in vitro* to induce the re-expression of genes putatively silenced by promoter methylation(1).

5-Aza-CdR substituted for cytosine during replication and is recognized by DNA methyltransferases (DNMTs)(2). This treatment ultimately depletes cellular stores of DNMTs and results in widespread genomic hypomethylation(3). In addition to reactivation of methylation-silenced tumor-suppressor genes, 5-aza-Cyd and 5-aza-dCyd are highly toxic in cultured cells(4, 5) and animals(6, 7).

DNA methylation of CpG dinucleotides is known to be mediated by at least three DNMTs, including DNMT3A, DNMT3B, and DNMT1. DNMT3A and DNMT3B are de novo methyltransferases that initiate the methylation process, whereas DNMT1, the maintenance methyltransferase, directs methylation of the newly synthesized strand complimentary to the hemimethylated DNA(8). Studies suggested that over-expression of DNMT1 and /or DNMT3B involved in tumorigenesis and development of most cancers(9-12), including hepatocellular carcinoma(13). The DNMT1 siRNA treatment led to a partial removal of DNA methylation from inactive promoter CpG islands, and restored the expression of tumor suppressor genes(14). Gene targeting experiments have shown that DNMT3B plays an important role in the hypermethylation of CpG islands in human cancers(15). Rhee(16) demonstrated that somatic cell knockouts of both DNMT3B and DNMT1 genes led to demethylation and re-expression of tumor suppressor genes in a colon cancer cell line. We used a combination of genetic (siRNA) and pharmacological (5-aza-2'-deoxycytidine) inhibitors of DNMTs to study the contribution of the DNMT isotypes to cancer-cell methylation. In the current study, we investigated the effects of 5-aza-2'-deoxycytidine alone and suppression of DNMT1 or DNMT3B on human hepatoma cell lines. We try to explore the possibility of treatment strategy to hepatocellular carcinoma (HCC) by modifying the aberrant methylation.

## Materials and Methods

### Cell Culture and 5-Aza-CdR Treatment

The human hapetocellular carcinoma cell line SMMC-7721 was obtained from the Cell Bank (Shanghai, China). Cell lines were maintained in RPIM1640 medium supplemented with 12% heat-inactivate NBS, 100 units/ml of penicillin, and 100 units /ml of streptomycin. Cells were cultured at 75 ml flask and treated in the next day with 100 uM 5-Aza-CdR (Sigma Chemical Co., St. Louis, MO) up to 4 days. The cultured medium was changed 3 days after treatment, and total RNA was extracted at day 3 from exponentially growing cultures.

### Preparation of siRNA of DNMT, a vector-based siRNA construct and transfection of DMMT RNAi construct to hepatocellular carcinoma cell line SMMC-7721

SiRNAs targeting DNMT1 or DNMT3B were designed and prepared as previous described(17, 18). The siRNA sequences against *DNMT1* were designed as sense and antisense oligonucleotides corresponding to nucleotide position 2,620-2,638 of human *DNMT1* (GenBank accession No. NM001379.1). The sequence of DNMT3B siRNA corresponds to nucleotide position 470-488 of *DNMT3B* (GenBank accession no. AF331857), there was no homology with other human genes was found by scanning the GenBank of NCBI using these siRNA.

The human hepatocellular carcinoma cell line SMMC-7721 (No. TCHu13 Cell Bank Shanghai, China) was maintained by serial passage in RPMI 1640 (Life Technologies, Inc., Rockville, MD) containing 10% heat-inactivated new born bovine serum, 100 U/ml penicillin and 100 mg/ml streptomycin, and incubated at 37°C, 5% CO_2_, 95% air using the standard tissue culture incubators. One day before transfection, cells were seeded in order that they were 30–50% confluent the next day. Cells were transfected with 1.5ug of DNMT1 or DNMT3B siRNA (pMT1 or pMT3B) construct using transfectamine transfection reagent (Invitrogen) reduced serum medium at 37°C in a 5% CO_2_ atmosphere for 5 h. The medium was removed and replaced with fresh RPMI 1640 supplemented with 20% new born bovine serum. Control cells were treated with transfectamine alone or with pSUPER-GFP plasmid. Cells were grown and selectively cultured in 0.4 mg/ml Genetincin (life technologies) for two months after the initial transfection. The cells stably harboring targeting vector were monitored with GFP expression. SMMC-7721 cells were transfected with pMT1 named as 7721-MT1 cell lines, with pMT3B named as 7721-MT3B cell lines.

### RNA extraction and synthesis of cDNA first strand

Total RNA was extracted using TRIZOL (Gibco BRL, Life Technologies Inc.,) according to the manufacturer’s protocol. RNA was resuspended in DEPC treated water and quality verified by agarose gel electrophoresis stained by EB. Total RNA was reverse transcribed using 2 µg of RNA and oligo-dT(18) (Life Technologies, Inc.) in a 13.5-ul reaction. The oligo-dT-RNA mixture was placed at 70°C for 2 min and then rapidly cooled to 0°C. Then, the Superscript II reverse transcriptase and its MIX (Life Technologies, Inc.) was supplied into the oligo-dT-RNA mixture followed by 42°C for 60 min, and 90°C for 5 min and then rapidly cooled to 0°C.

### cDNA microarrary

The 14K cDNA microarrary used in this study were obtained through the Shanghai BioChip Co., Ltd. The 14K slides contained human genes together with 10 positive and 6 negative controls. The spotting patterns and the complete annotated list of cDNAs are available at the following web site: http://www.shbiochip.com. The 5’ends of the genes up-regulated in this manner were analyzed in the BLAST database to determine whether they have promoter CpG islands.

## Results

### 5-Aza-CdR induced genes expression in global changes

We first assessed the global changes in gene expression induced by 5-Aza-CdR in the liver tumor cell line exposed to 100uM 5-Aza-CdR for 4 days. We analyzed those genes that were up- or down-regulated by more than 2.5-fold 4 days after drug treatment and categorized the genes as to whether or not they are known to be in some pathways. The 5’ ends of the genes up-regulated in this manner were analyzed in the BLAST database to determine whether they might have promoter CpG islands. The data showed that only 70% up-regulated genes in the hepatocellular carcinoma cell line definitely contained 5’ CpG islands.

### SiRNA of DNMT1 or DNMT3B constructs induced genes expression in global changes not only in those containing CpG island in the promoter regions

We next assessed the global changes in gene expression induced by siRNA of DNMT1 constructs or siRNA of DNMT3B constructs in hepetocellular carcinoma cell lines SMMC-7721. There is some information indicating that genes of non-CpG island promoter up-regulates expression induced by knockdown DNMT1 or DNMT3B, therefore, the mechanism by which the knockdown of DNMT1 or DNMT3B activated genes without CpG islands remains unclear.

### SiRNA of DNMT3B induces more genes identical to induced by 5-Aza-CdR than siRNA of DNMT1 in hepatocellular carcinoma cells

**Figure 1:**
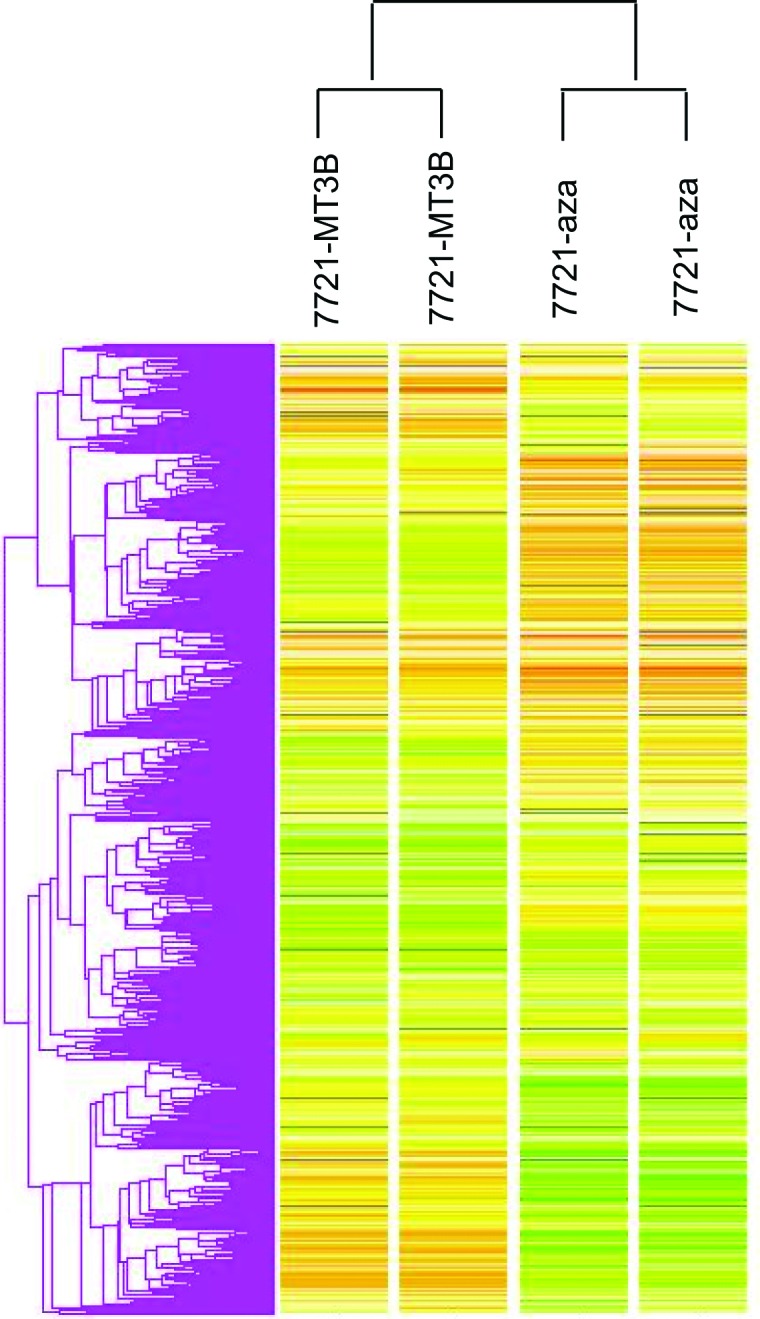
Hierarchical clustering based on gene expression data from SMMC-7721 DNMT3B unsuppressed and suppressed cells, and SMMC-7721 5-Aza-CdR untreated and treated cells.

We compared those genes that both were up-regulated by DNMT1 siRNA and 5-Aza-CdR treatment, and also compared up-regulated genes both were induced by DNMT3B siRNA and 5-Aza-CdR treatment. A total of 9 genes were found to be over expressed by more than two-fold induced by DNMT1 siRNA and 5-Aza-CdR in SMMC-7721 (Table 1). The 5’ ends of the genes up-regulated in this manner were analyzed in the BLAST database to determine whether they have promoter CpG islands. The data showed that almost all of up-regulated genes contained 5’ CpG islands except *KRT14*. We next focused our attention on those genes that induced by DNMT3B siRNA with demethylation drug. A total of 30 genes were found to be over expressed by more than two-fold induced by DNMT3B siRNA and 5-Aza-CdR in SMMC-7721. The 5’ ends of the genes up-regulated in this manner were analyzed in the BLAST database to determine whether they have promoter CpG islands. The data showed that 76.6% up-regulated genes conjectural contained 5' CpG islands (Table 2). Hierarchical clustering of gene expression in untreated and treated cells is shown in Fig. 1. As expected, some similarities in gene expression were seen between untreated cell by DNMT3B RNAi and 5-Aza-CdR.

**Table 1:** Genes altered more than 2-fold after 5-Aza-CdR and pMT1 treatment in the hepatocellular carcinoma SMMC-7721 cell line.

	GenBank_ID	Gene_Symbol	5’CpG island (CGI)^a^	Function
Regulation of transcription	NM_182917	*EIF4G1*	CGI	regulation of translational initiation
				
Signal transduction	NM_002733	*PRKAG1*	CGI	regulates adipose differentiation
	NM_004583	*RAB5C*	CGI	signal transduction, regulation
				
Others	NM_016219	*MAN1B1*	CGI	hydrolase activity
	NM_017883	*WDR13*	CGI	mRNA processing
	NM_000526	*KRT14*	None	epidermis development
	NM_004860	*FXR2*	CGI	insulin receptor binding
	NM_005993	*TBCD*	CGI	integrin-mediated signaling pathway
	NM_000034	*ALDOA*	CGI	RNA binding

a Based on a PubMed search; CGI, 5’ CpG island; None, no known 5’ CpG island.

**Table 2:** Genes altered by more than 2.5-fold after pMT3B and 5-Aza-CdR treatment in the hepatocellular carcinoma cell line SMMC-7721

GenBank_ID	Symbol	5 CpG island (CGI)^a^	Function
Cell differentiation			
NM_006096	NDRG1	CGI	cell differentiation
			
Cell proliferation			
NM_005557	KRT16	NO	cell proliferation
NM_013402	FADS1	CGI	regulation of cell differentiation
			
Regulation of transcription			
NM_003926	MBD3	CGI	regulation of transcription
NM_005253	FOSL2	CGI	regulation of transcription
			
Signal transduction			
NM_138769	ARHT2	CGI	signal transduction
NM_004218	RAB11B	CGI	signal transduction
NM_145173	DIRAS1	CGI	signal transduction
			
Others			
NM_017510	HSGP25L2G	CGI	intracellular protein transport
NM_006700	FLN29	CGI	nucleic acid binding
NM_001956	EDN2	NO	protein kinase C activation
NM_057089	AP1S1	CGI	intracellular protein transport
NM_020196	XAB2	CGI	DNA repair
NM_006412	AGPAT2	CGI	metabolism
NM_001074	UGT2B7	NO	metabolism
NM_006247	PPP5C	CGI	regulation of I-kappaB kinase/NF-kappaB cascade
NM_002273	KRT8	NO	cytoskeleton organization and biogenesis
NM_006445	PRPF8	CGI	nuclear mRNA splicing, via spliceosome
NM_002233	KCNA4	CGI	potassium ion transport
NM_004204	PIGQ	CGI	carbohydrate metabolism
NM_004818	DDX23	CGI	protein kinase C activation
NM_017458	MVP	NO	response to drug
NM_032038	SPINL	CGI	tetracycline transport
NM_020230	PPAN	CGI	RNA splicing
NM_001897	CSPG4	CGI	cell motility
NM_021979	HSPA2	NO	male meiosis
NM_018110	DOK4	CGI	insulin receptor binding
NM_144568	C14orf9	CGI	hydrolase activity

a Based on a PubMed search; CGI, 5’ CpG island; None, no known 5’ CpG island.

## Discussion

Epigenetic mechanisms including DNA methylation and histone modifications are one of the most important means of gene expression regulation. More and more evidences indicated that epigenetic modifications have a crucial role in cancer development. Epigenetic therapy is also a potential therapy because epigenetic defects are thought to be more easily reversible with pharmacological intervention(19, 20). Aberrant DNA hypermethylation, a prevalent alteration in most tumor playing a key role in human carcinogenesis, involved in global hypomethylation(21-23), specific gene hypermethylation(24-26) and loss of imprinting (LOI)(27, 28). DNMTs are responsible for setting up and maintenance of DNA methylation in biological process and thought to be involved in tumorigenesis. Therefore DNMTs theoretically serve as a reasonable target for anti-tumor therapy. In preclinical work, DNMT inhibitors have reversed the growth of cancer cell lines and demonstrated antineoplastic effects in animal models, including prolongation of survival(29, 30). One such agent, 5-aza-2’-deoxycytidine (5-aza-CdR), is a potent inhibitor of genomic and promoter-specific DNA methylation(25). However, there also are limitations of epigenetic therapeutic agents for prevention of tumorigenesis due to lack of specificity, these result in accelerated tumor progression(31, 32) and drug toxicity(33, 34). Moreover, it is necessary to find a better way to correct epigenetic abnormity in tumor, especially in DNA methylation.

Evidences showed that DNMT1 and DNMT3B cooperatively maintain DNA methylation and gene silencing in human cancer cells(16, 35). DNMT3B may not only silence genes by several mechanisms including direct DNA methylation or recruitment of proteins that modify chromatin, but also play an important role in transformation(36, 37). DNMT3B over-expression may be involved in the suppression or lower expression of p14ARF and p16INK4a observed in esophageal squamous cell carcinoma(38). The DNMT1 or DNMT 3B can be considered as a target for epigenetic therapy in human tumor. Antisense oligonucleotides are short, defined sequences of nucleotides that are complementary to mRNAs and hybridize with them and make them inactive, thereby blocking translation. Antisense oligonucleotides that are complementary to mRNA for human DNMT1 are undergoing preclinical(39) as well as clinical(40) trials. RNA interference (RNAi) is a natural mechanism in organisms in resistance to virus invasion and inhibition of transposon mobility by double stranded RNA (dsRNA).

Latest study shows that 21-25 nt small interference RNA (siRNA) can mediate specific gene silencing in mammal cells(41). Being effective and highly specific, RNAi probably becomes a novel technique in knocking gene down and plays important roles in gene function study and gene therapy of diseases(42). In this study, we try to knock down the expression of DNMT1 or DNMT3B via siRNA to explore the mechanism of DNMTs involved in hepatoma therapy. An advantage of using high-throughput oligonucleotide microarray data from treated cell lines is to enable us to provide a conservative estimate of the number of genes directly affected by aberrant methylation in hepatocellular carcinoma cell line. There are different induced genes by suppression of DNMTs among DNMT1 siRNA, DNMT3B siRNA and demehylation drug. Compared with inhibition of DNMT1, DNMT3B siRNA induced more tumor-related genes identical to that of demehylation drug 5-aza-2’-deoxycytidine.

Approximate 33.3% (10/30) genes were reported to be involved in tumorigenesis, such as *FADS1*, *MBD3*, *MVP*, *DIRAS1* and so on. Among them, there are 76.6% up-regulated genes conjectural contained 5’ CpG islands. These data indicated a new strategy in therapy of hepatocellular carcinoma through suppression of DNMT3B rather than treatment with demethylation agent. Because it has not been reported that DNMT3B are highly toxic in cultured cells and animals. Recently, frequent epigenetic changes such as DNA hypo- and hypermethylation and altered methylation pattern have been observed in hepatocellular carcinoma(43, 44). In fact, over-expression of DNMT3b4 is involved in human hepatocarcinogenesis, even at the precancerous stages by affecting the expression of specific genes(45). An increase in the DNMT3B mRNA levels in hepatocellular carcinoma relative to their non-cancerous tissues may be a predictor of poor survival(46). Over expression of a splice variant of DNMT 3B, DNMT3b4, is associated with DNA hypomethylation on pericentromeric satellite regions during human hepatocarcinogenesis(47). One of the novel findings of our present study is that DNMT3B siRNA could induce more genes identical to demethylation agent in hepatocellular cancer. Although these results need to be extended in larger clinical studies, this may serve as a potential new approach for hepatocellular carcinoma.
